# Assessment of Pharmacology, Safety, and Metabolic activity of Capsaicin Feeding in Mice

**DOI:** 10.1038/s41598-019-45050-0

**Published:** 2019-06-13

**Authors:** Padmamalini Baskaran, Laurel Markert, Jane Bennis, Liesl Zimmerman, Jonathan Fox, Baskaran Thyagarajan

**Affiliations:** 1 School of Pharmacy, University of Wyoming, Laramie, WY 82071 USA; 2 Department of Veterinary Sciences, University of Wyoming, Laramie, WY 82071 USA

**Keywords:** Obesity, Obesity

## Abstract

Capsaicin (CAP) activates transient receptor potential vanilloid subfamily 1 (TRPV1) to counter high-fat diet (HFD)-induced obesity. Several studies suggest that CAP induces the browning of white adipocytes *in vitro* or inguinal white adipose tissue (iWAT) *in vivo*. However, there is a lack of data on the dose-response for CAP to inhibit HFD-induced obesity. Therefore, we first performed experiments to correlate the effect of various doses of CAP to prevent HFD-induced weight gain in wild-type (WT) mice. Next, we performed a subchronic safety study in WT mice fed a normal chow diet (NCD ± CAP, 0.01% in NCD) or HFD ± CAP (0.01% in HFD) for eight months. We analyzed the expression of adipogenic and thermogenic genes and proteins in the iWAT from these mice, conducted histological studies of vital organs, measured the inflammatory cytokines in plasma and iWAT, and evaluated liver and kidney functions. The dose-response study showed that CAP, at doses above 0.001% in HFD, countered HFD-induced obesity in mice. However, no difference in the anti-obesity effect of CAP was observed at doses above 0.003% in HFD. Also, CAP, above 0.001%, enhanced the expression of sirtuin-1 and thermogenic uncoupling protein 1 (UCP-1) in the iWAT. Safety analyses suggest that CAP did not cause inflammation. However, HFD elevated plasma alanine aminotransferase and creatinine, caused iWAT hypertrophy and hepatic steatosis, and CAP reversed these. Our data suggest that CAP antagonizes HFD-induced metabolic stress and inflammation, while it does not cause any systemic toxicities and is well tolerated by mice.

## Introduction

Capsaicin (CAP) is a key ingredient of natural chili peppers. The benefits of CAP to treat neuropathic pain^1–3^, cancer^4–7^ and metabolic syndrome^8–14^ have been extensively researched in the recent years. Previous experimental studies using CAP patches applied locally on rats and rabbits suggest that CAP was safe and well tolerated^15^. Further, evaluation of external CAP patch to treat neuropathic pain suggests that CAP is safe and well tolerated by humans^16^. However, there is a lack of data on the long-term safety and effectiveness of oral feeding of CAP. CAP prevents obesity and enhances energy expenditure in rodents^17–21^ and humans^22–24^. CAP activates the beiging of inguinal white fat (WAT) to promote energy expenditure^10^ and enhances thermogenic program in brown fat to counter high fat diet (HFD)-induced obesity in mice^9^ without decreasing energy intake. Also, CAP counters hyperlipidemia and glucose intolerance and increases plasma glucagon like peptide 1 (GLP-1) level^9^. However, CAP did not prevent obesity in mice that genetically lacked the capsaicin receptor, transient receptor potential vanilloid subfamily 1 (TRPV1)^9,10^. Further, previously published studies have used several forms of capsaicin, either as chili pepper powder, its non-pungent analog capsiate, or other forms of capsaicinoids and reported variable outcomes on the safety of such compounds^25–42^. Such variations result from the type and purity of capsaicinoids or capsinoids used in these studies. Furthermore, a recent research suggests that pungent CAP is more efficient in binding to TRPV1 and enhance thermogenesis compared with non-pungent derivatives^43^. Also, most of these data from rodents were obtained using 0.01% of CAP in HFD and there is lack of a dose response correlation between the dose of CAP and the inhibition of HFD-induced weight gain.

Since there is lack of data on the pharmacological effect of oral feeding of various doses of pure CAP (not as red pepper powder, chili powder, its derivative, capsaicinoids or capsinoids) and its long-term safety in mice, we performed a dose response study and 8-month subchronic safety analyses by feeding mice with pure CAP either in normal chow diet (NCD) or HFD (60% calories from fat).

## Results

### Dose response for CAP on the inhibition of HFD-induced weight gain

**Figure 1:**
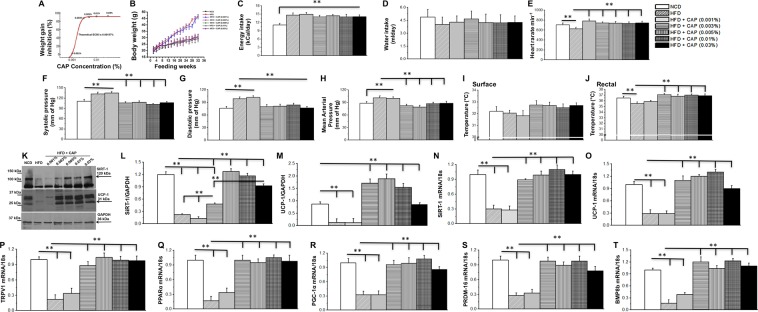
Dose response for CAP-induced suppression of HFD-mediated weight gain. (**A**) Effect of various concentration of CAP on HFD-induced weight gain in male WT mice. (**B**) Average weight gain in NCD or HFD (±CAP)-fed WT mice. (**C**,**D**) Average energy and water intake in WT mice-fed HFD (±CAP). Energy/water intake were measured in kCal/day and mL/day, respectively, and averaged for the entire duration of the study. Mean ± S.E.M of heat rate (**E**) systolic and diastolic blood pressure (**F**,**G**) mean arterial pressure (**H**) and surface and rectal body temperature (**I**,**J**) in HFD (±CAP)-fed mice (n = 8). (**K**) Western blot shows the expression of SiRT-1 and UCP-1 in the iWAT of NCD or HFD (±CAP, various concentrations) in WT mice, which received either NCD or HFD (±CAP) for 32 weeks. (**L**,**M**) The band intensity ratio for SiRT-1 and UCP-1 to GAPDH (loading control) ±S.E.M is given for n = 3 independent experiments. Mean mRNA levels ±S.E.M of SiRT-1 (**N**) UCP-1 (**O**) TRPV1 (**P**) PPARα (**Q**) PGC-1α (**R**) PRDM-16 (**S**) and BMP8b (**T**) in the inguinal WAT of WT mice-fed HFD (±CAP, various concentrations; n = 8/condition and experiments were performed in triplicates). **Represent statistical significance for P < 0.01 between either NCD and HFD-fed groups or HFD and HFD + CAP-fed groups.

Surprisingly, overwhelming number of previously published works have used CAP at a concentration of 0.01% in diet. There is lack of information on the effect of CAP at lower concentrations to inhibit HFD-induced obesity. To establish a correlation between various doses of CAP and inhibition of HFD (60% calories from fat)-induced obesity, we fed WT mice (8 mice/group) a HFD (± 0.001%, 0.003%, 0.005%, 0.01% or 0.03% of CAP, which correspond to a 0.133 mg/kg, 0.399 mg/kg, 0.665 mg/kg, 1.33 mg/kg and 3.99 mg/kg body weight) for 32 weeks (from week 6 through 38). All doses of CAP, above 0.001% in HFD, significantly inhibited weight gain in wild type (WT) mice (Fig. 1A). Also, the difference among the efficacy of various doses of CAP to counter weight gain was insignificant (Fig. 1B). Also, CAP feeding did not alter energy or water intake in WT mice (Fig. 1C,D). To evaluate whether CAP reverses HFD-induced hypertension, we measured the heart rate, blood pressure, and mean arterial pressure in HFD (±CAP)-fed mice (Fig. 1E through H). HFD significantly decreased heart rate (Fig. 1E), increased systolic and diastolic blood pressure and mean arterial pressure (Fig. 1F–H) and CAP countered these. Also, we measured the body temperature of these mice (Fig. 1I,J). As shown in Fig. 1J, HFD decreased the rectal temperature of mice and CAP reversed these at doses above 0.001% in HFD. However, CAP (0.001%) did not reverse the inhibitory effect of HFD on blood pressure. The mean weight of vital organs and tissues of NCD or HFD (±CAP, various doses) are summarized in Tables 1 and 2. Although CAP profoundly inhibited HFD-induced weight gain at a dose as low as 0.003% in HFD, we chose to use a higher dose (0.01%) to perform safety studies in WT mice. Demonstrating the safety of CAP at this dose in mouse via a subchronic eight months study is important for advancing its druggability against diet-induced obesity in humans.

**Table 1:** The mean organ/tissue weights normalized to total body weight for NCD (±0.01%) CAP and HFD (±CAP)-fed wild type mice.

Organ/tissue (g/g)	NCD	NCD + 0.01% CAP	HFD	HFD + 0.001% CAP	HFD + 0.003% CAP	HFD + 0.005% CAP	HFD + 0.01% CAP	HFD + 0.03% CAP
Heart	0.006 ± 0.001	0.007 ± 0.0003	0.003 ± 0.0002^$$,**^	0.003 ± 0.0001^$$,**^	0.006 ± 0.0003	0.008 ± 0.001	0.005 ± 0.0005	0.005 ± 0.001
Liver	0.057 ± 0.002	0.049 ± 0.003	0.045 ± 0.002^$$,**^	0.048 ± 0.002^$$,**^	0.042 ± 0.002	0.036 ± 0.005	0.042 ± 0.001	0.041 ± 0.002
Kidneys	0.014 ± 0.001	0.013 ± 0.0005	0.004 ± 0.0005^$$,**^	0.003 ± 0.001^$$,**^	0.011 ± 0.0005	0.014 ± 0.001	0.007 ± 0.0005	0.01 ± 0.001
Spleen	0.003 ± 0.0002	0.004 ± 0.0001	0.002 ± 0.0001^$$,**^	0.002 ± 0.0001^$$,**^	0.004 ± 0.001	0.004 ± 0.001	0.001 ± 0.0005	0.003 ± 0.0005
Epididymal fat	0.016 ± 0.001	0.015 ± 0.001	0.032 ± 0.004^$$,**^	0.035 ± 0.002^$$,**^	0.038 ± 0.007	0.027 ± 0.004	0.029 ± 0.003	0.053 ± 0.008
Inguinal	0.008 ± 0.001	0.01 ± 0.0005	0.049 ± 0.003^$$,**^	0.05 ± 0.005^$$,**^	0.024 ± 0.003	0.019 ± 0.003	0.019 ± 0.002	0.022 ± 0.004
Brown fat	0.005 ± 0.001	0.007 ± 0.001	0.011 ± 0.001^$$,**^	0.014 ± 0.002^$$,**^	0.013 ± 0.001	0.007 ± 0.002	0.004 ± 0.0004	0.012 ± 0.001
Retroperitoneal fat	0.008 ± 0.004	0.004 ± 0.001	0.023 ± 0.002^$$,**^	0.03 ± 0.001^$$,**^	0.016 ± 0.003	0.013 ± 0.004	0.012 ± 0.002	0.015 ± 0.003

^$$^Significance for P < 0.01 between NCD and HFD (±0.001% CAP) groups. **Significance for P < 0.01 between HFD (±0.001% CAP) and HFD (±CAP >0.001%) groups.

**Table 2:** The mean organ/tissue weights ±S.E.M. normalized to tibial length for NCD (±0.01%) CAP and HFD (±CAP)-fed wild type mice.

Organ/tissue (g/cm)	NCD	NCD + 0.01% CAP	HFD	HFD + 0.001% CAP	HFD + 0.003% CAP	HFD + 0.005% CAP	HFD + 0.01% CAP	HFD + 0.03% CAP
Heart	0.11 ± 0.01	0.11 ± 0.01	0.11 ± 0.004	0.10 ± 0.003	0.12 ± 0.01	0.11 ± 0.01	0.09 ± 0.004	0.1 ± 0.01
Liver	0.94 ± 0.05	0.81 ± 0.06	1.21 ± 0.06	1.24 ± 0.04	0.75 ± 0.05	0.79 ± 0.07	0.73 ± 0.03	0.84 ± 0.04
Kidneys	0.24 ± 0.01	0.21 ± 0.02	0.28 ± 0.02	0.3 ± 0.03	0.21 ± 0.01	0.19 ± 0.01	0.22 ± 0.01	0.21 ± 0.02
Spleen	0.05 ± 0.004	0.06 ± 0.004	0.08 ± 0.004	0.085 ± 0.002	0.08 ± 0.02	0.06 ± 0.01	0.05 ± 0.004	0.07 ± 0.01
Epididymal fat	0.27 ± 0.03	0.25 ± 0.03	0.87 ± 0.11	0.89 ± 0.14	0.76 ± 0.19	0.37 ± 0.06	0.53 ± 0.07	1.12 ± 0.21
Inguinal	0.15 ± 0.01	0.16 ± 0.01	1.26 ± 0.08	1.22 ± 0.02	0.46 ± 0.09	0.25 ± 0.03	0.34 ± 0.05	0.45 ± 0.1
Brown fat	0.08 ± 0.01	0.11 ± 0.02	0.28 ± 0.02	0.3 ± 0.03	0.25 ± 0.05	0.18 ± 0.03	0.17 ± 0.04	0.25 ± 0.03
Retroperitoneal fat	0.13 ± 0.06	0.07 ± 0.01	0.65 ± 0.05^$$,**^	0.70 ± 0.04^$$,**^	0.29 ± 0.07	0.18 ± 0.05	0.22 ± 0.04	0.33 ± 0.08

^$$^Significance for P < 0.01 between NCD and HFD (±0.001% CAP) groups. **Significance for P < 0.01 between HFD (±0.001% CAP) and HFD (±CAP >0.001%) groups.

### HFD-suppresses thermogenic gene expression and CAP counters this

Previous research suggests that HFD suppresses TRPV1 expression in WAT and BAT and CAP reversed this^9,10^. Also, CAP feeding enhances the expression of sirtuin-1 (SiRT-1; an NAD+-dependent deacetylase, which regulates cellular metabolism) and induces the browning of WAT by enhancing UCP-1 expression^9,10^. Therefore, we examined whether CAP, at all doses, enhance the expression of SiRT-1 and UCP-1 in inguinal WAT (iWAT). As shown in Fig. 1K–M, CAP increased the expression of both SiRT-1 and UCP-1 at doses above 0.001%. Further, HFD significantly decreased the mRNA levels of SiRT-1, UCP-1, TRPV1, peroxisome proliferator activated receptor alpha (PPARα), PPAR gamma coactivator 1 alpha (PGC-1α), PR domain containing protein 16 (PRDM-16), and bone morphogenetic protein 8b (BMP8b) in inguinal WAT and CAP reversed this at doses above 0.001% (Fig. 1N through T).

### CAP counters obesity in female WT mice

**Figure 2:**
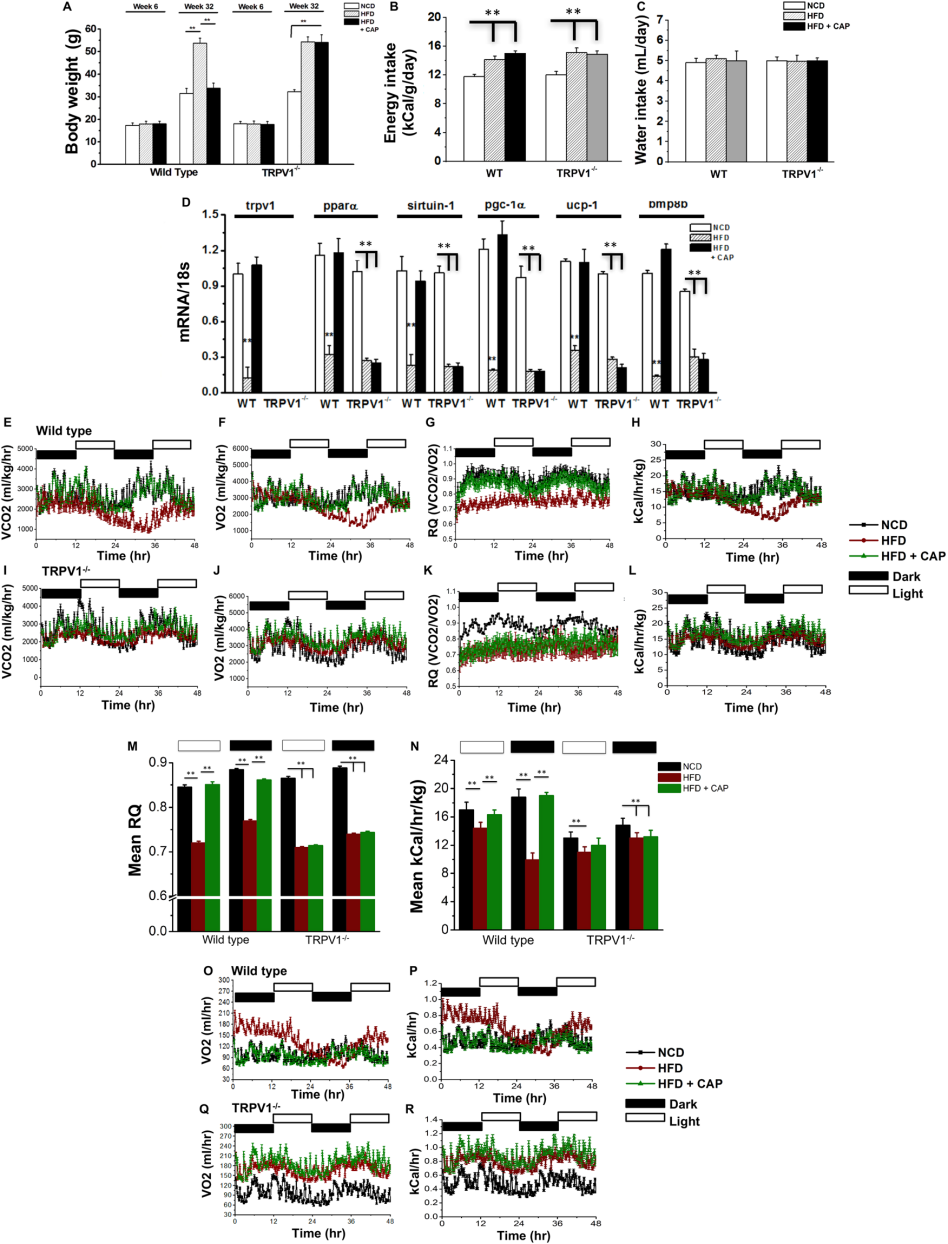
CAP counters HFD-induced obesity in female WT mice. (**A**) Body weight gain at week 6 and week 32 in NCD or HFD (±CAP)-fed WT and TRPV1^−/−^ mice. (**B**,**C**) Average food and water intake, respectively, in these mice. Food/water intake were measured in kCal/day and mL/day and averaged for the entire duration of the study. (**D**) Relative expression of adipogenic and thermogenic genes in the inguinal WAT of these mice at 32 weeks of feeding the respective diet. (n = 8 mice per condition and experiments were performed in triplicates). (**E**) through (**L**). Representative traces of VCO_2_, VO_2_, RER (respiratory quotient, ratio between VCO_2_ and VO_2_) 32 weeks of feeding NCD or HFD (± CAP) in the light and dark cycle. (**M**,**N**) Mean RER and energy expenditure ±S.E.M. for n = 4 per condition. The VO_2_ and energy expenditure for the wild type (**O**,**P**) and TRPV1^−/−^ (**Q**,**R**) mice without body weights. **Represent statistical significance for P < 0.01 between either NCD and HFD-fed groups or HFD and HFD + CAP-fed groups.

Since limited knowledge exists on the ability of CAP to inhibit obesity in female mice, we set to evaluate the effect of feeding HFD (±0.01% CAP) in WT and TRPV1^−/−^ female mice. As illustrated in Fig. 2A, CAP countered HFD-induced weight gain in the WT but not TRPV1^−/−^ female mice. CAP did not decrease the average daily energy or water intake in these mice (Fig. 2B,C). Furthermore, HFD suppressed the expression of TRPV1, PPARα, SiRT-1, PGC-1α, UCP-1 and BMP8b in the iWAT of these mice and CAP remarkably countered this (Fig. 2D). Next, we performed indirect calorimetry in NCD or HFD (±CAP)-fed WT and TRPV1^−/−^ female mice to determine the effect of CAP on respiratory quotient and energy expenditure. Representative graphs for VCO_2_, VO_2_, respiratory quotient (RQ = VCO_2_/VO_2_) and energy expenditure are given for wild type and TRPV1^−/−^ mice with respect to body weight in Fig. 2E through L. The mean RER and energy expenditure ±S.E.M. are given in Fig. 2M,N. The VO_2_ and energy expenditure without taking into account the body weight of the mice are given in Fig. 2O through R. CAP increased the RER. The energy expenditure was low in HFD-fed obese mice when calculated with respect to body weight. HFD + CAP-fed mice had the same energy expenditure as that of NCD-fed mice. But the energy expenditure was high in the obese mice when expressed as kCal/hour indicating that a unit of fat mass has a lower level of oxygen consumption thereby energy expenditure than a unit of lean mass. Similar to this, TRPV1^−/−^ mice fed with HFD ± CAP had low energy expenditure when calculated with respect to body weight and higher energy expenditure when calculated as kCal/hour without body weight.

**Figure 3:**
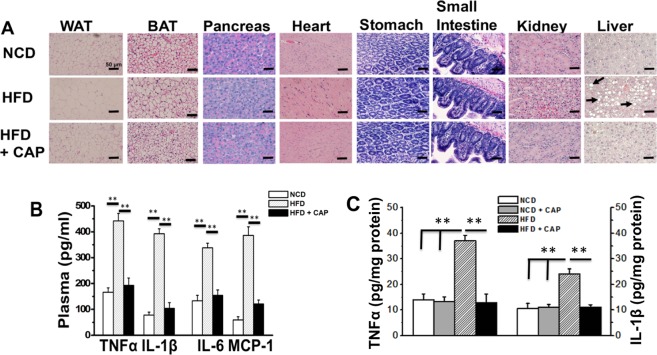
Histology sections of tissues of HFD (±CAP)-fed WT mice. (**A**) Histological architecture in paraffin sections of tissues from NCD or HFD (±0.01% CAP)-fed male WT mice (n = 8 per condition). Scale bar represents 50 μm. (**B**) Mean plasma levels (pg/ml) ±S.E.M. of TNFα, IL-1β, IL-6 and MCP-1 in WT mice (n = 4 experiments). (**C**) Cytokine levels in inguinal WAT of WT mice (n = 4 experiments). **Represent statistical significance for P < 0.01 between either NCD and HFD-fed groups or HFD and HFD + CAP-fed groups.

We isolated vital organs from NCD or HFD (±0.01% CAP)-fed WT mice and performed paraffin sections to analyze the morphology of these tissues. A histopathologist, who was blinded to the experimental group, performed these analyses. HFD caused iWAT and BAT hypertrophy and increased steatosis in the liver tissue, which CAP countered (Fig. 3A). Also, CAP did not cause any inflammation in any of the vital tissues. We also analyzed the expression of inflammatory cytokines in the iWAT and BAT of these mice. As shown in Fig. 3B, HFD increased the inflammatory markers in the plasma of wild type mice and CAP reversed it. More significantly, CAP reversed the effect of HFD on serum alanine aminotransferase and creatinine in the WT mice at doses above 0.001% in HFD (Table 3). Also, we measured the levels of Tumor necrosis factor 1 alpha (TNFα) and interleukin 1 beta (IL-1β) in the inguinal WAT of NCD (±CAP) or HFD (±CAP)-fed mice. CAP suppressed the levels of these inflammatory cytokines only in the inguinal WAT of HFD-fed mice.

**Table 3:** Clinical chemistry of HFD (±CAP)-fed WT mice.

Parameters Measured	NCD	HFD	HFD + CAP (%)				
			0.001	0.003	0.005	0.01	0.03
Total plasma protein (g/dl)	4.8 ± 0.33	5.3 ± 0.59	4.96 ± 0.49	4.81 ± 0.22	4.91 ± 0.21	4.79 ± 0.15	4.89 ± 0.05
Alkaline phosphatase (U/L)	55 ± 7.17	55.5 ± 9.07	62.66 ± 6.31	40 ± 4.42	36 ± 3.07	40 ± 5.03	64 ± 4.13
Alanine aminotransferase (ALT; U/L)	39 ± 3.64	101 ± 10.88^$$,**^	98.33 ± 8.56^$$,**^	36 ± 2.51	39 ± 3.05	40.3 ± 4.12	35 ± 5.22
Lipase (for acute pancreatitis; U/L)	125 ± 16.2	120 ± 10.11	115.88 ± 9.92	107.3 ± 15.6	96.14 ± 7.11	125 ± 5.09	96.51 ± 4.91
Creatinine (Kidney function test; mg/dl)	0.4 ± 0.14	1.1 ± 0.26^$$,**^	1.01 ± ×0.091^$$,**^	0.2 ± 0.05	0.2 ± 0.12	0.3 ± 0.04	0.4 ± 0.03
Blood Urea Nitrogen (BUN; mg/dl)	33.6 ± 0.72	26.2 ± 2.81	28.23 ± 1.08	23 ± 0.51	25 ± 0.05	32 ± 0.51	25 ± 0.23
WBC count/μL	7.4 ± 1.3	6.1 ± 0.26	6.2 ± 0.22	6.4 ± 1.24	5.9 ± 1.52	6.4 ± 1.32	6.1 ± 1.57
Hemoglobin (g/dl)	13.5 ± 1.10	14.2 ± 1.52	13.88 ± 1.26	13.2 ± 1.24	13.6 ± 1.56	14.2 ± 1.13	13.9 ± 1.21
Erythrocytes ×10^6^/μL	8.64 ± 1.08	9.21 ± 1.11	8.89 ± 1.01	8.43 ± 1.52	8.97 ± 1.21	9.47 ± 1.04	9.2 ± 1.22
Platelets ×10^3^/μL	797 ± 112	722 ± 100	766 ± 114	711 ± 150	714 ± 112	736 ± 120	725 ± 106

^$$^Significance for P < 0.01 between NCD and HFD (±0.001% CAP) groups.

**Significance for P < 0.01 between HFD (±0.001% CAP) and HFD (±CAP >0.001%) groups.

### CAP neither alters weight gain and nor modifies energy intake in NCD-fed mice

CAP is a pungent compound from chili peppers. When it binds to its receptor on the sensory nerve endings in the oral cavity, it causes burning and irritation of the mucous membranes. Although several research works demonstrate the beneficial effects of CAP against pain, arthritis, obesity, cardiovascular diseases and cancer, conflicting studies suggesting the deleterious effects of CAP do exist. Therefore, we evaluated the effect of feeding 0.01% CAP in NCD in the WT mice and determined the body weight gain, energy intake and metabolic activity. We chose a dose of 0.01% of pure CAP in NCD for the safety experiments since at this dose CAP significantly inhibited HFD-induced obesity. Since previous studies have not evaluated the effect of long-term feeding of CAP at the dose or 0.01% in NCD, we fed mice either NCD or NCD (±0.01 CAP) for 32 weeks, isolated the inguinal WAT at the end of 32 weeks to analyze the expression of thermogenic UCP-1 and SiRT-1.

**Figure 4:**
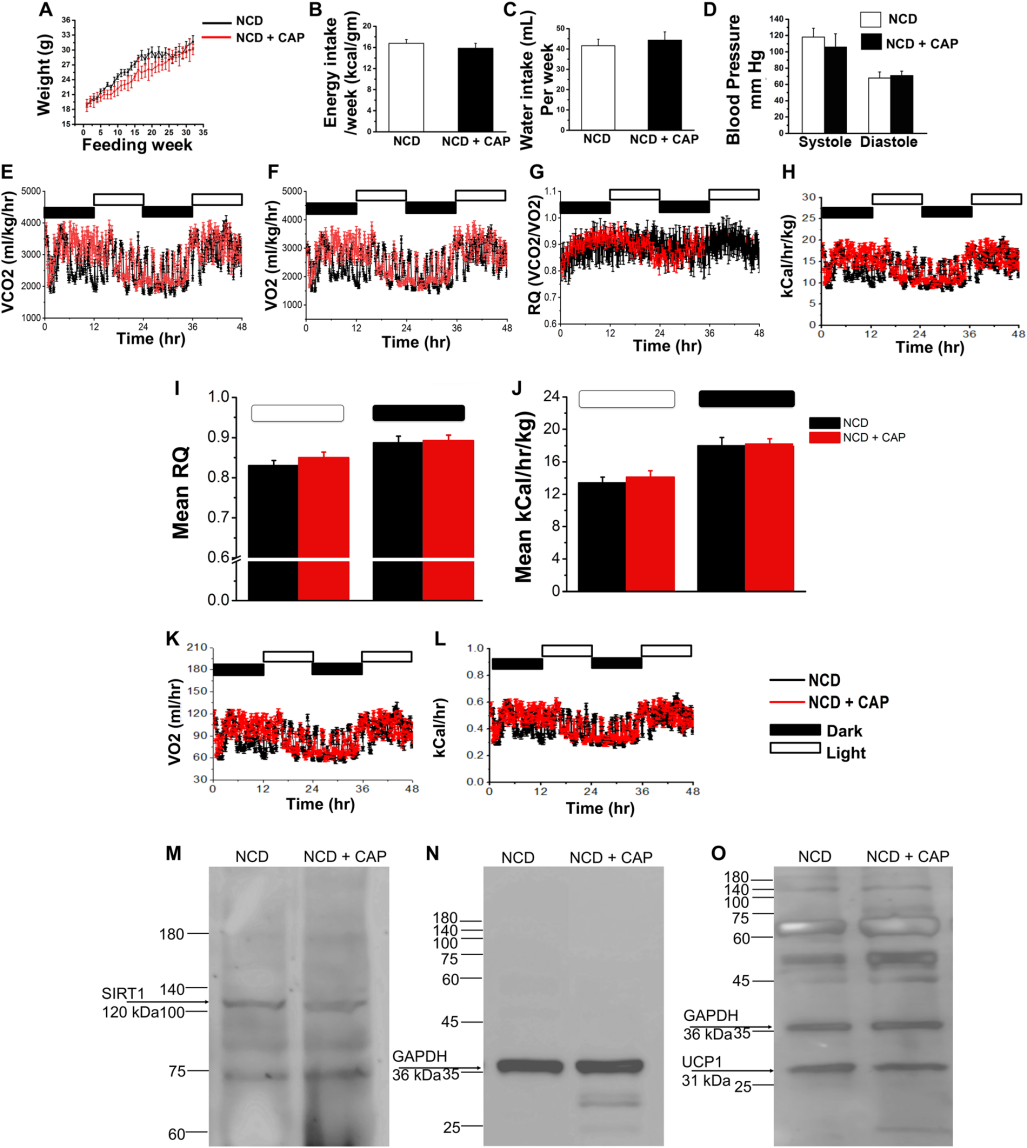
Effect of CAP on body weight and metabolic parameters in NCD-fed WT mice. (**A**) Time course of weight gain in NCD (±CAP)-fed mice. (**B**,**C**) Mean energy and water intake/day ±S.E.M. (**D**) Mean blood pressure ±S.E.M. in NCD (±CAP)-fed WT mice. (**E**) through (**H**). Representative traces of VCO_2_, VO_2_, RER (respiratory quotient, ratio between VCO_2_ and VO_2_) 32 weeks of feeding NCD (±CAP) in the light and dark cycle. (**I**,**J**) Mean RER and energy expenditure ±S.E.M. for n = 4 per condition. The VO_2_ and energy expenditure for the wild type (**K**,**L**). Representative western blot showing the expression of SiRT-1 (**M**), GAPDH (**O**; loading control), and UCP-1 (**O**) in the inguinal WAT of NCD (±CAP)-fed mice.

We fed sub-groups of WT mice a NCD (±CAP) for 8 months. WT mice received these diets from week 6 onwards and until week 38. As illustrated in Fig. 4A–C, CAP neither prevented weight gain nor altered food and water intake in NCD-fed WT mice. CAP did not alter the fasting cholesterol, blood glucose, weight of vital organs, (Tables 1 and 2) or blood pressure (Fig. 4D) in these mice. We also analyzed the RQ and energy expenditure (heat) in these mice at 32 weeks of feeding these diets. As shown in Figure 42E through L, we did not observe any change in these parameters between NCD (±CAP)-fed mice. The RQ and energy expenditure for these mice are shown in Fig. 4E though J with respect to body weight and without body weight (Fig. 4K,L) for these mice. Since CAP countered the suppression of thermogenic SiRT-1 and UCP-1 by HFD in the inguinal WAT, we analyzed the expression of these proteins in the inguinal WAT of NCD (±CAP)-fed mice. Representative western blots for the expression of SiRT-1 and UCP-1 are shown in Fig. 4M through O. GAPDH was a loading control. Also, CAP did not alter the hemodynamic parameters and blood chemistry in NCD-fed mice (Tables 3 and 4).

**Table 4:** Clinical chemistry of NCD (± CAP)-fed WT mice.

Parameters Measured	NCD (n = 6)	NCD + CAP (n = 6)
Total plasma protein (g/dl)	4.8 ± 0.3	5.1 ± 0.2
Alkaline phosphatase (U/l)	60 ± 4.9	80 ± 3.3
Alanine aminotransferase (U/l)	39 ± 3.6	30 ± 1.8
Lipase (U/l)	125 ± 16.2	83.2 ± 11.7
Creatinine (Kidney function test) (mg/dl)	0.4 ± 0.1	0.35 ± 0.08
Blood Urea Nitrogen (mg/dl)	33.6 ± 0.7	28.7 ± 0.9
WBC count (per μl)	7.4 ± 1.3	9.3 ± 1.2
Hemoglobin (g/dl)	13.5 ± 1.1	12.6 ± 1.08
Erythrocytes (per μl)	8.64 ± 1.08 × 10^6^	9.25 ± 0.96 × 10^6^
Platelets (per μl)	797 ± 112 × 10^3^	837 ± 108 × 10^3^
Fasting glucose (mg/dl)	88 ± 12.5	90.4 ± 8.7
Fasting cholesterol (mg/dl)	93 ± 13	72 ± 7

### CAP does not alter cardiac functions in WT mice

**Figure 5:**
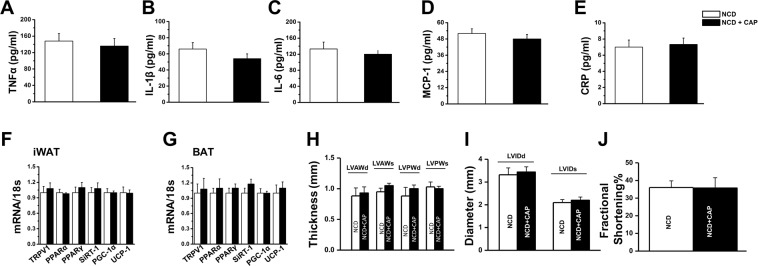
Effect of CAP on plasma inflammatory cytokines and echo cardiac parameters in NCD-fed WT mice. Plasma concentrations of TNFα (**A**) IL-1β (**B**) IL-6 (**C**) MCP-1 (**D**) and CRP (**E**) in NCD (±CAP)-fed WT mice (n = 8). Mean mRNA levels ±S.E.M of TRPV1, PPARα, PPARγ, SiRT-1, PGC-1α and UCP-1 in the inguinal WAT (iWAT; **F**) and BAT (**G**) of WT mice-fed NCD (±CAP) for 32 weeks (n = 8). (**H**) Mean left ventricular anterior and posterior wall thickness ±S.E.M. during diastole and systole. (**I**) Mean left ventricular internal diameter ±S.E.M. during diastole and systole. (**J**) Mean Fractional shortening % ±S.E.M. in NCD (±CAP)-fed mice (n = 8).

Next, we performed echocardiography for WT mice-fed NCD (±CAP) and measured the thickness of left ventricular anterior and posterior wall (Fig. 5H) and left ventricular internal diameter (Fig. 5I) during diastole and systole and fractional shortening (Fig. 5J). CAP did not alter any of these values.

**Figure 6:**
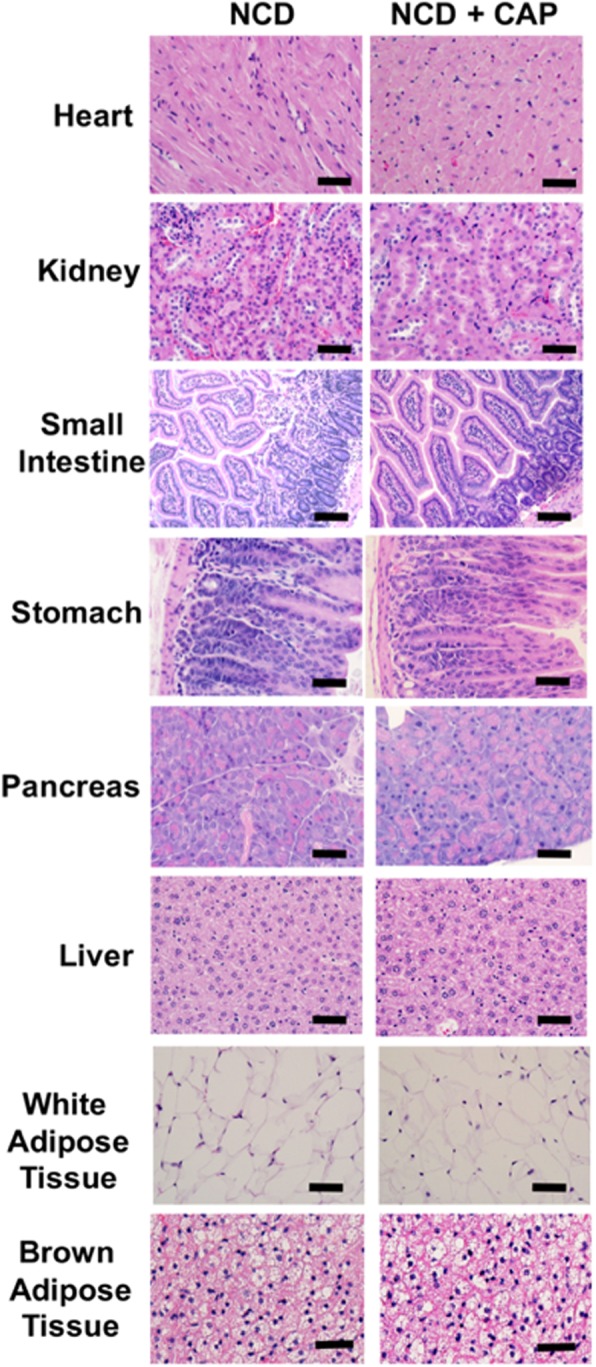
CAP does not result in microscopic lesions following subchronic dosing. Histological architecture in paraffin sections of tissues from NCD (±0.01% CAP)-fed male WT mice. Differences between the two groups were not found (n = 8). Scale bar represents 50 μm.

To determine whether CAP causes inflammation, we measured the plasma levels of inflammatory cytokines in NCD (±CAP)-fed mice. As shown in Fig. 5A through E, CAP feeding did not cause any increase in the plasma levels of TNFα, IL-1β, IL-6, monocyte chemoattractant protein 1 (MCP-1) or C reactive protein (CRP) in the WT mice. In NCD-fed WT mice, CAP did not alter the mRNA levels of TRPV1, PPARα, PPARγ, SiRT-1, PGC-1α or UCP-1 in inguinal WAT (iWAT) and BAT (Fig. 5F,G). We also prepared paraffin sections of vital tissues obtained from NCD (±CAP)-fed WT mice. The histology sections shown in Fig. 6 suggest that CAP did not cause any inflammation in vital tissues when fed orally for 8-months.

### CAP does not alter neuromuscular coordination in WT mice

**Figure 7:**
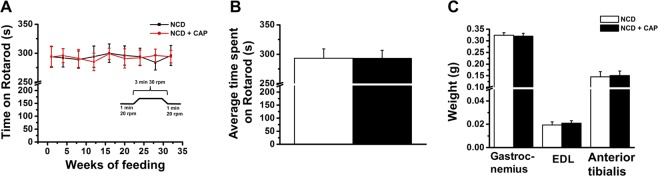
CAP does not affect neuromuscular coordination and performance in WT mice. (**A**) Time spent on a rotarod by WT mice fed NCD (±CAP) for 32 weeks. (**B**) The average time spent on rotarod for the entire duration of the study (32 weeks) by WT mice-fed NCD (±CAP). (**C**) The mean weight (±S.E.M.) for gastrocnemius, *extensor digitorum longus* (EDL) and *anterior tibialis* skeletal muscles in WT mice fed NCD (±CAP) for 32 weeks (n = 8 per condition).

Previously published work suggests that CAP injection in the hindlimbs of mice prevented the neuroparalytic effect of botulinum neurotoxin A^34^. However, no deleterious effect was observed in the control mice when CAP was injected *in vivo*^34^. In order to analyze whether long-term oral CAP administration alters neuromuscular coordination, we determined the effect of oral feeding of NCD (±CAP) on the performance of WT mice on a rotarod. We analyzed the ability of mice to walk on a rotating rod during the 32 weeks of feeding. Also, we measured the dry weight of gastrocnemius, *extensor digitorum longus* and *tibialis anterior* muscles at the end of 32 weeks of feeding NCD (±CAP) to analyze whether the subchronic CAP feeding alters muscle weight. As explained in the Fig. 7, CAP neither affected the performance of WT mice on rotator nor altered the dry weight of skeletal muscles.

**Figure 8:**
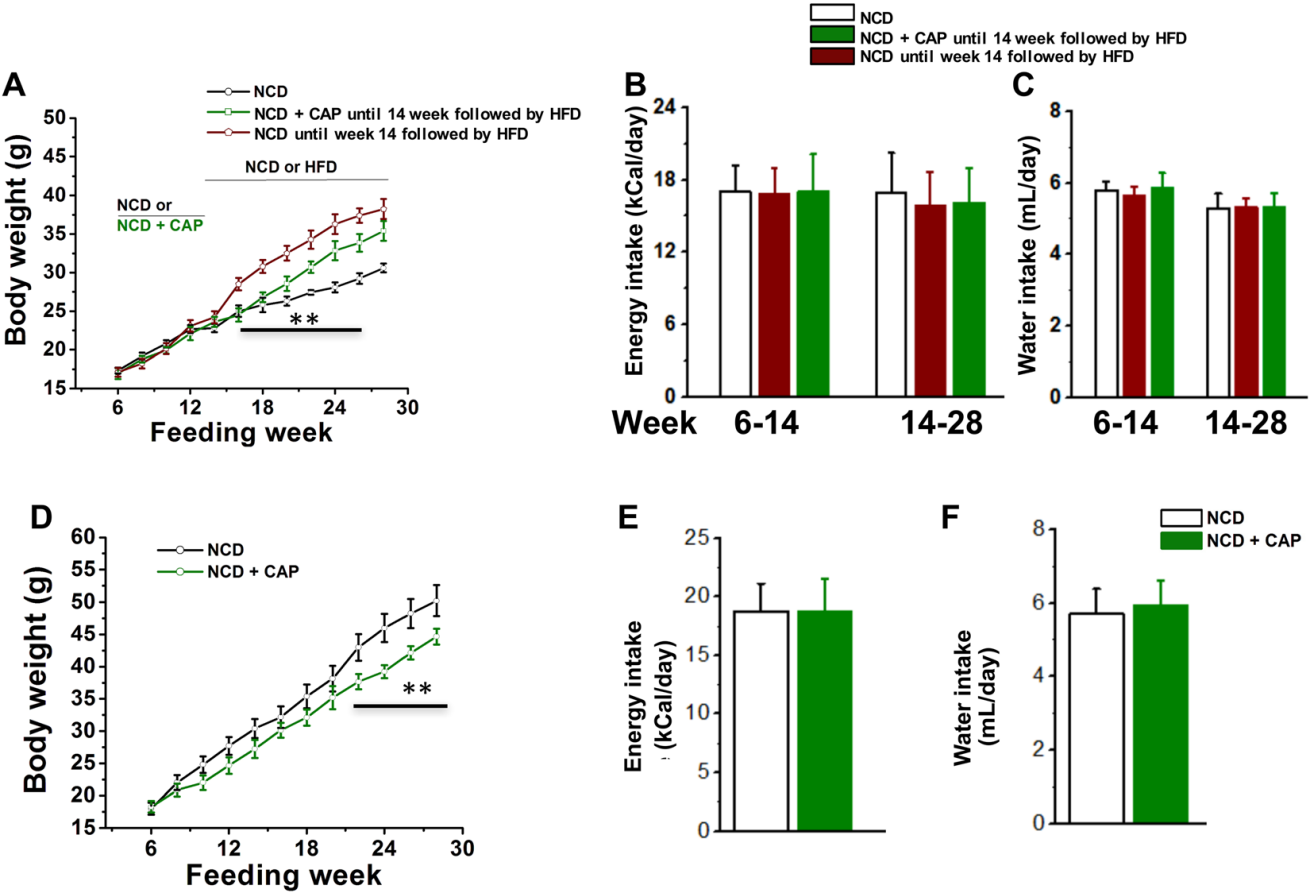
CAP prevents weight gain in a dynamic and genetic model of obesity. (**A**) Body weight in WT mice that received NCD (black and wine) or NCD + CAP (olive) for 14 weeks and then switched to a NCD (black) or HFD (wine) after week 14. (**B**,**C**) Average daily energy and water intake ±S.E.M. in these mice. (**D**) Body weight in NCD (±CAP)-fed Ob/Ob mice. (**E**,**F**) Average daily energy and water intake in NCD (±CAP)-fed Ob/Ob mice. ** represents statistical significance for P < 0.01 between either NCD and HFD or HFD and HFD + CAP fed groups in A and between NCD and NCD + CAP groups in C for n = 4 mice under each condition. The black line indicates the significance between the body weight of the groups that received HFD following NCD or NCD + CAP.

The preferential effect of CAP to antagonize HFD-induced metabolic stress and the lack of effect of CAP on NCD-fed WT mice suggest that the beneficial effect of CAP is metabolic stress dependent. To analyze this, we fed WT mice either NCD or NCD + CAP for 14 weeks and then switched to a HFD for 14 more weeks. A sub-group of mice that continued to receive NCD served as controls. As shown in Fig. 8A, CAP did not alter weight gain when fed with NCD. However, HFD-induced weight gain was significantly less in mice that received CAP + NCD for 14 weeks. Further, energy intake or water consumption was not different in these mice (Fig. 8B,C).

To evaluate whether CAP dynamically exerts its anti-obesity effect in genetic model of obesity, we fed Ob/Ob mice a NCD (±CAP) for 28 weeks. That is, Ob/Ob mice received NCD until 6 weeks of age and then either continued NCD or NCD [±0.01% CAP until week 34 (28 weeks of feeding)]. Also, we measured the energy and water intake in these mice. As described in Fig. 8D, CAP did not alter weight gain in Ob/Ob mice until feeding week 20. However, it caused a slight but significant suppression of weight gain in these mice compared to NCD-fed controls, without altering energy/water intake in these mice (Fig. 8E,F).

## Discussion

Previous research works have shown that TRPV1 activation by CAP is important for its anti-obesity action. This study shows that CAP inhibits HFD-induced obesity in both male and female mice. Consistently, published works from our laboratory unequivocally suggest that CAP prevents HFD (D12492; 60% calories from fat)-induced obesity in the wild type mice but not in TRPV1^−/−^ mice^9,10,43^. However, a previously published work suggests that lack of TRPV1 prevents obesity diet (28.5% calories from fat)-induced obesity in mice^44^. In contrast to this observation, a recent study indicates that lack of TRPV1 accelerates diet (Harlan Teklad TD93075; 55% calories from fat)-induced obesity in mice^45^. The different types of diets used in these studies, which may differ significantly in their micro and macronutrient contents, the amount and types of fat used could contribute to such variabilities in the results. observed.

Published studies have used various doses of CAP, ranging from 1.33 mg/kg body weight to 161.2 mg/kg body weight, in the form of capsinoids and capsaicinoids for efficacy, safety or toxicity studies in rodents. More importantly, the various types of CAP, like chili pepper powder, cayenne or even non-pungent capsiate, were used in these studies. Further, several laboratories have shown the potency of CAP and its analogs in preventing diet-induced obesity in mammals^13,19,45–48^. However, long-term safety and efficacy data are not available for pure CAP. In this study, we performed a dose response for CAP to inhibit HFD-induced obesity in WT mice. Also, we thoroughly evaluated the safety of CAP at a higher dose that inhibited HFD-induced obesity in mice.

The results from this subchronic 8-month feeding study with a standardized dose of pure capsaicin did not observe any toxicity of feeding CAP. This suggests that CAP is safe when fed at concentrations ranging between 0.003 and 0.03% in the diet. The dose response relationship suggests that all doses of CAP, except the lowest dose of 0.001% (corresponds to 0.133 mg/kg body weight) in HFD, inhibited HFD-induced weight gain. We did not observe any significant differences in the weight gain inhibition among other doses of CAP greater than 0.001% in HFD. Our study presents a theoretical IC50 of 0.00157% CAP to inhibit HFD-induced obesity. At a dose of 0.01% in HFD, CAP significantly prevented hypertrophy of WAT and BAT and suppressed steatosis in HFD-fed mice. This was associated with a protection of liver and kidney functions by CAP in HFD-fed WT mice at doses above 0.001% (Table 3). It is surprising that we did not observe a clear dose response on the inhibitory effect of CAP, since all concentrations, above 0.001% in HFD, inhibited weight gain in mice. Also, these concentrations CAP (except 0.001%) significantly enhanced the expression of thermogenic SiRT-1 and UCP-1 in the iWAT. This indicates that CAP has very high potency in enhancing these thermogenic proteins to counter obesity. The ability of CAP to enhance SiRT-1 activity to promote the browning phenomenon in WAT and UCP-1-mediated thermogenesis are important for the anti-obesity effect of CAP. Possibly, CAP enhances fatty acid oxidation and energy expenditure to mediate its effects^20^.

Another important aspect of our study is that CAP suppresses HFD-induced obesity independent of gender difference. Our study indicates that CAP could prevent obesity by enhancing thermogenes expression in female WT mice but not in female TRPV1^−/−^ mice. The effect of CAP to prevent HFD-induced obesity in WT mice that received CAP in NCD until week 14 suggests that the anti-obesity effect of CAP existed even when CAP was discontinued in the diet. But, this difference did not last post week 25. Therefore, long-term studies are required to analyze how long the protective effect of CAP will last. Such studies should also address the effect of sustained and controlled release formulations of CAP, since such formulations may yield a longer effect of CAP in the body. Also, CAP feeding prevented weight gain in Ob/Ob mice but this effect was small but significant compared to its effect in WT mice. It is important to note that we could observe a small effect of CAP only after week 20 in NCD-fed Ob/Ob mice. This could be due to decreased expression level of TRPV1 in the iWAT of Ob/Ob mice. This notion is consistent with a previous research, which reported that TRPV1 expression is decreased in the visceral adipose tissue of Ob/Ob and db/db mice^19^. Consistent to our observation, a recent study suggests that CAP did not prevent weight gain in Ob/Ob mice when fed for a shorter period of 6 weeks but improved glucose handling in these mice^49^.

Published literature suggests that feeding CAP or red pepper with meal increased satiety due to pungency^39,40^. However, this study did not observe any effect of CAP on total energy intake in WT mice when fed either in NCD or HFD. Even though HFD feeding may decrease the pungency of CAP, the lack of effect of CAP on the amount of NCD consumed by mice suggest that the mice did not avoid CAP containing diet is surprising. This is consistent with previously published research^9,10,17,19^. Also, future studies are required to evaluate whether CAP affects the diurnal pattern of energy intake in rodents.

The lack of effect of CAP on dietary intake in these mice also raises an important question whether CAP increases the circulating levels of orexigenic (ghrelin) and anorexigenic (glucagon like peptide-1). Published work suggest that HFD suppressed circulating GLP-1 and CAP countered this in the wild type but not TRPV1^−/−^ mice^9^. However, CAP did not suppress energy intake or appetite in these mice^9,10,21,43^. Consistent with this observation administration of CAP in diet failed to decrease ghrelin in humans while increased GLP-1^50^. Another study in humans suggests that CAP increased GLP-1 and decreased ghrelin but had not effect on energy intake^51^. Several studies suggest that CAP decreases circulating ghrelin^51^. Also, chemical vagal de-afferentiation with capsaicin blocked the ability of peripherally administer ghrelin to stimulate food intake^52^. However, several published studies indicate that CAP did not decrease energy intake in rodents but countered diet-induced obesity^9,10,19,21,43,53^. These data highlight a disconnect between the effect of CAP in suppressing appetite to mediate its anti-obesity effect. However, it is important to note that the dose and the type of CAP may play a role in its appetite suppressive effect. Further, considering the short half-life of GLP-1 in the circulation, whether CAP caused a sustained elevation GLP-1 to mediate its effect on appetite control is not known. Further studies are required to analyze these effects of CAP.

**Figure 9:**
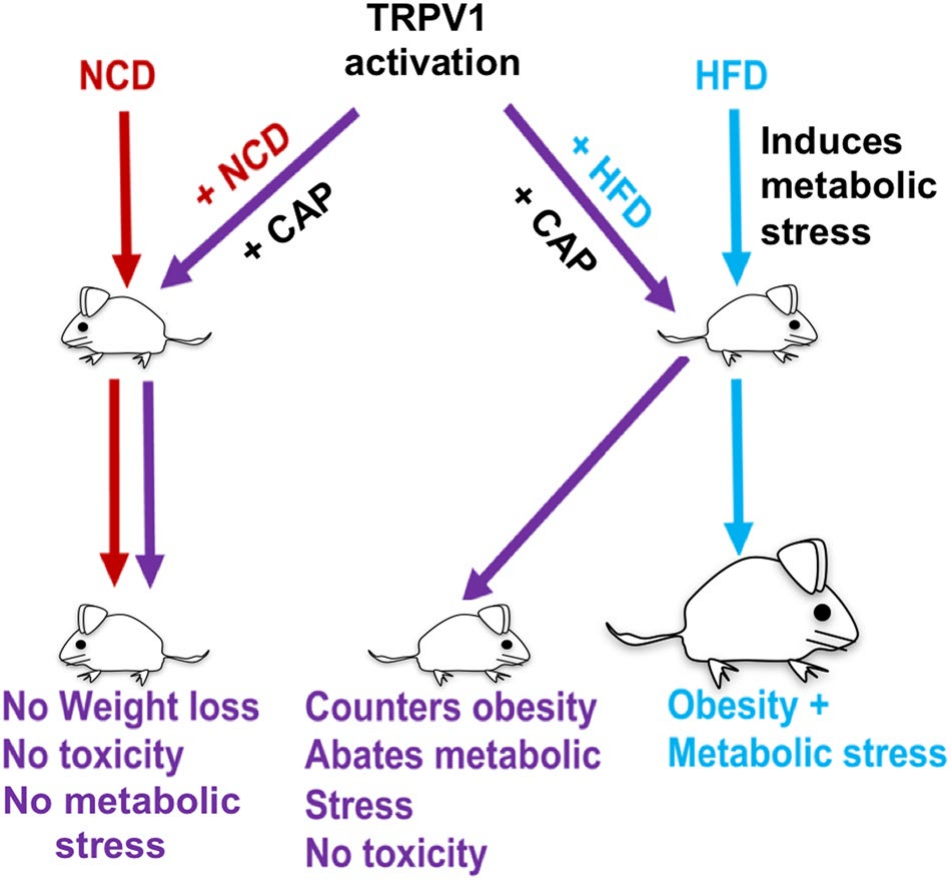
TRPV1 activation prevents HFD-induced metabolic stress and does not alter physiological functions in the absence of metabolic stress. Chronic feeding of CAP does not affect any physiological parameters but significantly inhibits metabolic pathologies. HFD induces a metabolic stress that promotes obesity, hyperlipidemia, hypertension and hepatic steatosis. CAP counters this and enhances the expression of thermogenic genes/proteins.

Our study shows that CAP did not inhibit body weight gain in the NCD-fed group (Fig. 4A), while suppressed weight gain in HFD-fed mice. Further, the lack of effect of CAP on the mRNA levels of adipogenic genes and thermogenic genes in NCD-fed group suggests that the metabolically beneficial effects of CAP depend on metabolic stress induced by HFD. Previous works also suggest that CAP reversed the heat production, respiratory quotient (RER, respiratory exchange ratio) and locomotion activity in HFD-fed WT mice^9,10,21^. However, CAP did not affect heat and RER under NCD-fed condition (Fig. 4G–I). This suggests that in the absence of a HFD-induced metabolic stress, CAP does not cause any alterations in metabolic activity, weight gain or toxicities (model shown in Fig. 9). That is, CAP fails to exert a thermogenic effect in the absence of a metabolic stress. But, the mechanism behind this observation remains unclear. Further studies are required to understand this. Also, future studies should verify whether such an effect of CAP can be observed in the presence of stress factors, other than HFD, that cause metabolic dysfunction.

Further, the lack of toxicity of CAP in the histology of vital tissues in NCD-fed WT mice suggests that oral CAP is safe and well tolerated by mice. Moreover, the long-term, 8-month, feeding of CAP failed to cause any changes in the neuromuscular coordination and cardiac properties.

Obesity is an inflammatory disease and increases the adipose and circulating levels of inflammatory cytokines^49,54–56^. Consistently, HFD enhanced the levels of inflammatory cytokines in the blood and inguinal WAT and CAP reversed this. However, long-term 8-months feeding of CAP did not cause any rise in the circulating levels of inflammatory markers such as (tumor necrosis factor 1 alpha (TNF-α), interleukin 1 beta (IL-1β), IL-6, MCP-1, and CRP (Fig. 5) in NCD-fed WT mice. Interestingly, unlike under HFD-fed conditions, CAP did not enhance the expression of thermogenic SiRT-1 and UCP-1 in NCD-fed mice. This data suggests that in the absence of a metabolic stress like HFD-induced obesity, CAP-induced activation of TRPV1 neither counters weight gain nor thermogenic protein expression in the WAT. Further, when fed with NCD, CAP did not alter the hematological and biochemical parameters described in Table 4. All together, these data suggest that CAP feeding did not cause any adverse effects in WT mice and was well tolerated at a dose of 0.01% in NCD. The ability of CAP to antagonize inflammation in HFD-fed mice indicates that it exerts its effect only in the presence of a metabolic stress and CAP *per se* does not induce inflammation or stress when fed with NCD.

Collectively, our research provides valuable data on the long-term safety of CAP. These data illustrate that CAP does not induce metabolic disturbance like increase in blood pressure or inflammation in mice but abates HFD-induced obesity and oxidative stress in mice. Specifically, when HFD-causes a metabolic stress, CAP is able to antagonize it without causing any side effects. Our subchronic safety study suggests that CAP is well tolerated and safe in mice. However, long-term genotoxicity studies still remain to be performed in WT mice to substantiate our findings. Further studies in humans to evaluate the safety and efficacy of CAP are warranted to promote the oral use of CAP as a novel anti-obesity agent.

## Materials and Methods

### Mouse model of HFD-induced obesity

**Figure 10:**
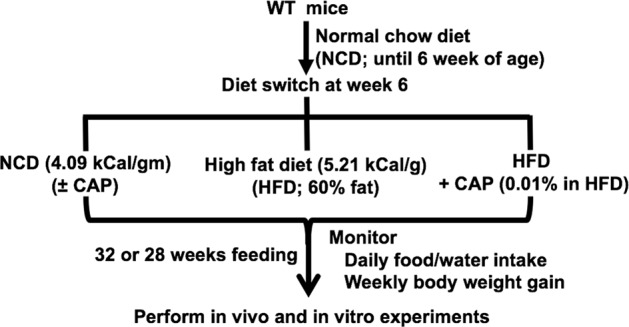
Methodology describing the study plan.

All animal protocols were approved by the Institutional Animal Care and Use Committee of the University of Wyoming (Protocol #20170119BT00262). Adult male and female TRPV1^−/−^ (Stock No. 003770) and genetically unaltered control wild type (WT) mice purchased from Jackson Laboratory, Maine, USA, were housed in groups of four in the research animal facility and maintained at 22–23 °C at the School of Pharmacy, University of Wyoming. Breeding colonies were maintained in the research animal facility of the School of Pharmacy, University of Wyoming. Wild type littermates were used as controls for the experiments. Proper husbandry care was followed as per the recommendation of the Institutional Animal Care and Use Committee of the University of Wyoming. Mice were allowed access to NCD, HFD (60% calories from fat; D12492; Research Diets, Inc., NJ) or HFD + CAP (various concentrations in HFD) diets and water *ad libitum*. The plan for feeding and experiments is described in Fig. 10. The calorific value of NCD and HFD are 4.09 kCal/g (Lab Diet 5001; www.labdiet.com, USA calories provided in % protein-28.672, fat 13.384 and carbohydrates 57.944) and 5.24 kCal/g (D12492; Research Diets Inc., NJ; calories provided by in % protein 20, fat 60 and carbohydrate 20) respectively.

Mice were fed normal chow diet (NCD) until 6 weeks after birth. Mice (n = 8 per group) were randomly assigned and housed in groups of four in separate cages and their weekly weight gain and food/water intake were recorded for analysis. The study design and animal ethics conformed to the recent guidance on experimental design and analysis and are reported in compliance with the ARRIVE guidelines^57,58^. Starting from week 6, wild type and TRPV1^−/−^ mice were randomly assigned into four groups and fed either NCD^59^, NCD + 0.01% CAP, HFD^60^ alone or HFD + CAP (different doses of CAP, 0.001%, 0.003%, 0.005%, 0.01% or 0.03%, in HFD) until week 32. All mice were allowed access to diet and water *ad libitum*. The average weekly weight gain and food/water intake were monitored and the weighing personnel was blinded on the groups of mice that received NCD or HFD (±CAP). All *in vivo* and *in vitro* data analyses using the mice and tissues obtained from the mice were blinded.

### Histology sections

Following the euthanization of mice as per approved IACUC protocols, the isolated tissues were fixed in 10% formalin solution. Fixed tissues were processed, using routine procedures, through graded ethanol solutions, into xylene then were embedded in paraffin. The paraffinized tissues where then sectioned at 8 microns, mounted onto glass slides, then deparaffinized through xylene then ethanol solutions. Finally, the hydrated sections were stained with hematoxylin and eosin prior to a final step of dehydration then coverslipped using permount®.

### Clinical chemistry

Blood was collected by retro-orbital bleeding from mice fed-NCD (±CAP) or HFD (±CAP) for 32 weeks into red-capped vacutainer tubes. The collected blood was incubated on ice for 30 min. and then centrifuged at 3000 rpm for 30 minutes to collect the serum. Alternatively, whole blood was collected in to lavender top EDTA sprayed vacutainers (BD Biosciences, USA). Measurements were made at the Wyoming Veterinary Science Laboratory, which is accredited by the American Association of Veterinary Laboratory Diagnosticians, within 24 hours.

### ELISA measurements

The iWAT was isolated from the mice at the end of the study, placed in micro centrifuge tubes, flash frozen in liquid nitrogen and stored at −80 °C until further processing for cytokine analysis. For experiment, tissues were thawed on ice, and 1 ml phosphate-buffered saline (PBS) containing protease inhibitors was added to the frozen tissue to avoid degradation on thawing. Subsequently the tissues were homogenized 3 times 15 sec at 4 °C using tissue homogenizer. The lysates were centrifuged at 164 × g, 4 °C, 10 minutes, sequentially for 2 times and the supernatants were analyzed for cytokines. Measurements of inflammatory cytokines in plasma and inguinal fat lysate were performed using commercially available ELISA kits (Biovision, USA) as per the manufacturers’ protocol using a TECAN plate reader.

### Metabolic study

Metabolic activity was determined by using the Comprehensive Laboratory Animal Monitoring System {CLAMS™, Columbus Instruments, Columbus, OH, USA}^61^. Mice were individually placed in the CLAMS metabolic cages with *ad libitum* access to food and water. After acclimatization for 24 hr., metabolic parameters including the volume of carbon dioxide produced (VCO_2_), the volume of oxygen consumed (VO_2_), the respiratory exchange ratio (RER = VCO_2_/VO_2_), the energy expenditure as caloric (heat) value and ambulatory activity were determined for 48 hr. Energy expenditure was calculated by using modified Weir equation^62^; {Metabolic rate = (3.941 * VO_2_ + 1.106 * VCO_2_)/100]}^63^. The locomotor activity of mice was analyzed by beam break analysis. The movement of the mice in X, Y and Z axes was averaged per hour and represented as beam breaks/min^9,10^.

### Blood pressure measurement

Arterial pressure was measured noninvasively *via* tail-cuff instrument and their recording system. The recordings of method and data analysis have been described previously^64^. Mean arterial pressure and heart rate data were collected between 8 and 11 a.m. and analyzed.

### Noninvasive echocardiography

Cardiac geometry and function were evaluated in anesthetized mice using a 2-dimensional (2-D) guided M-mode echocardiography (Phillips Sonos 5500) equipped with a 15–6 MHz linear transducer (Phillips Medical Systems, Andover, MD). The heart was imaged in the 2-D mode in the parasternal long-axis view with a depth setting of 2 cm. The M-mode cursor was positioned perpendicular to interventricular septum and posterior wall of left ventricle (LV) at the level of papillary muscles from the 2-D mode. The sweep speed was 100 mm/s for the M-mode. Diastolic wall thickness, end diastolic dimension (EDD) and end systolic dimension (ESD) were measured. All measurements were done from leading edge to leading edge in accordance with the Guidelines of the American Society of Echocardiography. The percentage of LV fractional shortening was calculated as [(EDD − ESD)/EDD] × 100^65^.

### Fasting glucose and cholesterol measurement

Following 12 hr. fasting period, blood glucose levels were measured by using a glucometer (Counter Next monitoring system and strips, Bayer, USA). Fasting serum cholesterol levels were measured using a commercially available kit (Biovision, USA).

### Body temperature measurement

Surface and rectal temperature of mice were measured using a IRB153 portable infrared thermometer and RET-3 rectal probe for mice (Bioseb.com, USA). Surface and rectal temperatures were measured in triplicates for each mouse for three consecutive days and the average was calculated and plotted.

### Rotarod experiment

Mice were trained on a rotarod (Columbus Instruments, Columbus, OH) for one week prior to the initiation of the respective diet. Mice were exercised by walking on the rotarod for a period of 5 min. The total duration of the protocol was divided into a 1 min. walk at 20 rpm, 3 min walk at 30 rpm and then a 1 min walk at 20 rpm. The upward and down ramp from 20 to 30 to 20 rpm occurred within the 3-min duration. Mice were exercised for 5 days a week for the entire duration of the feeding 32-weeks of study.

### Quantitative real-time polymerase chain reaction (qRT-PCR) measurements

Inguinal WAT (iWAT) was collected from NCD (±CAP) or HFD (±CAP)-fed mice as per previously published procedure^60^, and were used for quantitative RT-PCR experiments^9,10^. Total RNA from tissues was isolated using Tri-reagent (Sigma, USA) according to manufacturer’s instructions and cDNA was synthesized using Quantitect reverse transcription kit (Qiagen, Valencia, CA) using Q5plex PCR system (Qiagen Valencia, CA). Real -time PCR was performed using Quantitect SYBR green PCR kit on Q5plex system. No template control and a sample without reverse transcriptase were used as negative controls for every gene of interest. 18s mRNA was used as the reference gene. Amplification was performed using 20-µl-reaction volume according to manufacturer’s instruction. Each experiment was performed in triplicates for statistical analysis. The following qRT-PCR primers (IDT, USA) were used for the quantification of genes.

*18s* forward-5′-accgcagctaggaataatgga-3′; reverse-5′-gcctcagttccgaaaacca-3′;

*mtrpv1* forward-5′-caacaagaaggggcttacacc-3′; reverse-5′-tctggagaatgtaggccaagac-3′;

*pparα* forward-5′-gtaccactacggagttcacgcat-3′; reverse-5′-cgccgaaagaagcccttac-3′;

*sirt-1* forwardf-5′*-*tcgtggagacatttttaatcagg-3′; reverse-5′-gcttcatgatggcaagtgg-3′;

*pgc-1α* forward-5′-agagaggcagaagcagaaagcaat-3′; reverse-5′-attctgtccgcgttgtgtcagg-3′;

*ucp-1* forward-5′-cgactcagtccaagagtacttctcttc-3′; reverse-5′-gccggctgagatcttgtttc-3′;

*prdm-16*-5′-cagcacggtgaagccattc-3′; reverse-5′-gcgtgcatccgcttgtg-3′;

*bmp8b* forward-5′-tccaccaaccacgccactat-3′; reverse-5′-cagtaggcacacagcacacct-3′.

### Immunoblotting

iWAT were isolated from NCD (±CAP) or HFD (±CAP)-fed mice as described earlier^60^. Fat pads were washed with chilled PBS and homogenized in a lysis buffer (50 mM Tris pH 7.5, 250 mM sodium chloride, 0.5% NP40, 0.5% sodium deoxycholate, 2 mM EDTA, 0.5 mM dithiothreitol, 1 mM sodium orthovanadate and protease inhibitor cocktail) and centrifuged at 14000 rpm for 20 min at 4 °C and the supernatant was collected in a pre-chilled micro centrifuge tube. It was centrifuged again at 14000 rpm for 20 min at 4 °C to remove fat floating on the top. The supernatant was collected, aliquoted and flash frozen in liquid nitrogen and stored at −80 °C until use. The protein concentration of whole tissue lysate was then determined using Bradford method, and equal amounts of protein (40 μg) were separated by SDS-PAGE, transferred to nitrocellulose membrane and immunoblotted with specific antibodies against SiRT-1 (SC-74465), UCP-1 (SC-28766) and GAPDH (365062)^9,10^. Western blots were performed in triplicates and band intensity was calculated using image J software.

### Chemicals and drugs

All chemicals and reagents were obtained from Sigma, USA. High fat diet (D12492) was from Research Diets, Inc. NJ, USA. QRT-PCR primers were obtained from IDT, USA and antibodies were from Santa Cruz Biotechnology, USA.

### Statistical analyses

All data are expressed as means ± S.E.M. Comparisons between groups were analyzed using one-way ANOVA and post hoc analyses were done using Tukey test. We performed two-way ANOVA using Microsoft Excel Analysis Tool Pack to compare the inhibitory effects of various doses of CAP on HFD-induced obesity. By this method we also analyzed whether there was a significant difference among the effects of CAP at doses greater than 0.001% on the inhibition of HFD-induced obesity. A *P* value < 0.05 was considered as statistically significant. All other analyses were performed using Microcal Origin 9.0 software and figures were generated using the same program and then converted into image files using Adobe Photoshop software.

## Supplementary information

Supplementary Dataset 1
