# Supplementary material for: Assessment of Pharmacology, Safety, and Metabolic activity of Capsaicin Feeding in Mice
Source: Sci Rep. 2019 Jun 13;9:8588. doi: 10.1038/s41598-019-45050-0 (PMC6565628; doi:10.1038/s41598-019-45050-0)

Supplementary dataset – for Figure 1K

Assessment of Pharmacology, Safety and Metabolic activity of Capsaicin Feeding in Mice

Padmamalini Baskaran*, Laurel Markert*, Jane Bennis*, Liesl Zimmerman*, Jonathan Fox$ and Baskaran Thyagarajan*

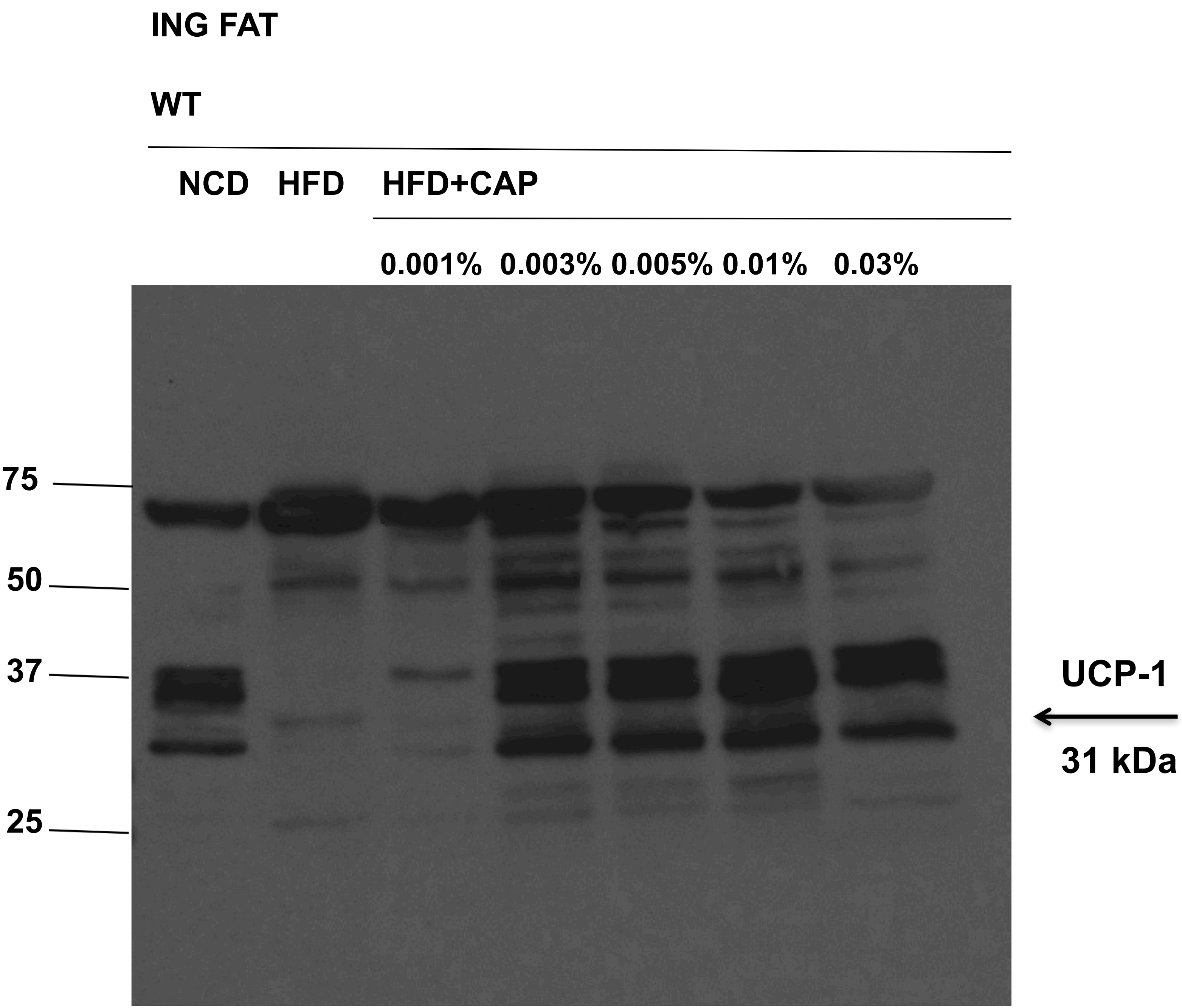
ING FAT
WT
NCD
HFD
HFD+CAP
0.001%
0.003%
0.005%
0.01%
0.03%
75
50
37
25
UCP-1
31 kDa

SIRT-1 bands deveoloped using Femto chemiluminescent reagent

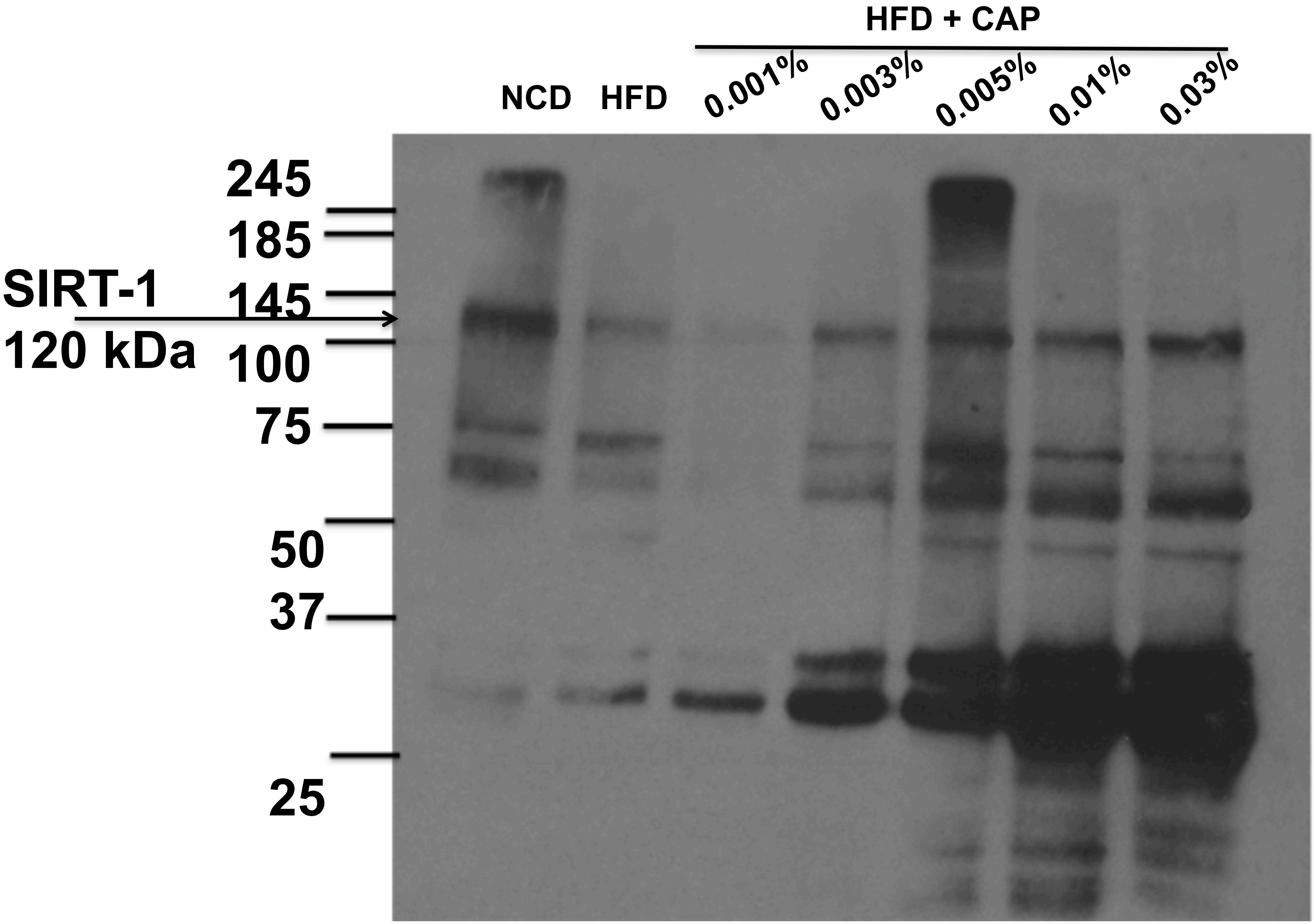

GAPDH was reprobed using western chemiluminescent reagent

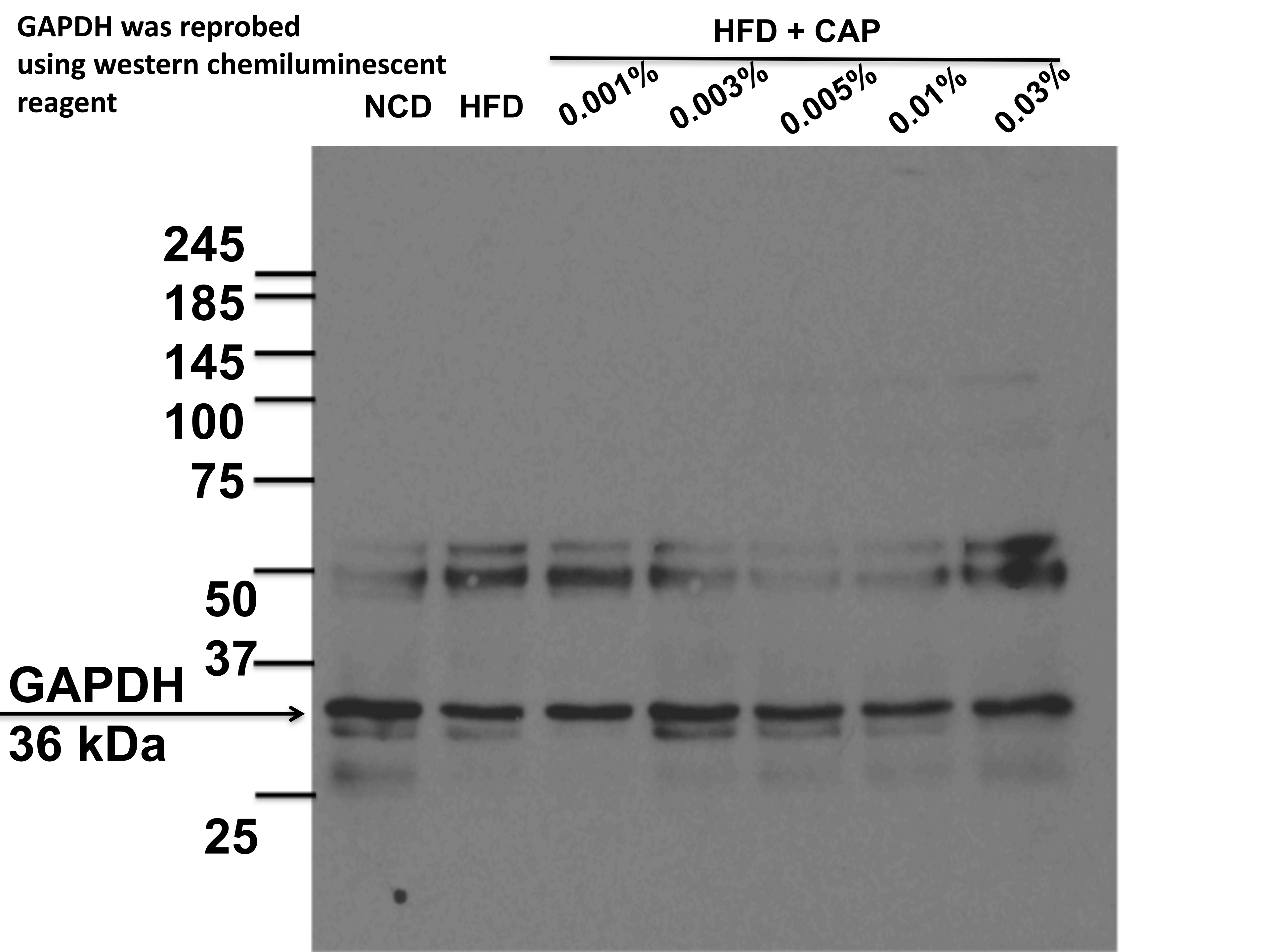

Supplement: Supplementary file 1 — Supplementary Dataset 1 [file 41598_2019_45050_MOESM1_ESM.pdf]
